# Single Visit versus Multiple Visit Root Canal Therapy

**DOI:** 10.5005/jp-journals-10005-1004

**Published:** 2008-12-26

**Authors:** Rajesh Singla, Nikhil Marwah, Samir Dutta

**Affiliations:** 1 Postgraduate Student, Govt. Dental College, Rohtak, India; 2 Asst. Professor, Govt. Dental College, Rohtak, India; 3 Sr. Professor and Head, Govt. Dental College, Rohtak, India

**Keywords:** single sitting, multiple sitting pulpectomy

## Abstract

*Aim:* The purpose of this study was to determine clinical success rate of single visit verses multiple visit root canal treatment in cariously exposed vital primary molars.

*Material& methods:* 40 children in age group of 4 to 7 years were divided equally into two treatment groups and recall visits were carried out after one week, one month and three months and six months.

*Results:* Statistically no significant difference was found.

Conclusion: Multiple visit and single visit root canal treatment demonstrated almost equal success but most important aspect for success in pulpectomy cases is the indication of each case and then its subsequent treatment, be it multiple or single visit root canal treatment.

## INTRODUCTION

In general, attitudes and concepts concerning proven, time tested treatment procedures are very slow and difficult to change in the health professions. We are often reluctant to abandon predictable treatment procedures because we fear a change to some new treatment modality may not result in the some outcome or take of success we have come to expect but as new data that may alter a pre-existing old concepts may have to expand in order to accommodate this new information. Research studies into intracanal preparation culturing, intracanal medicaments, and root canal filling materials and techniques have led us to expand and in some cases, completely alter our concepts concerning the clinical conduct of our practice in these areas.

Historically root canal treatment was performed in multiple visits mainly to ensure sterility of root canal system prior to obturation. As complete sterilization was not possible with biomechanical preparation and irrigation, intracanal medicaments were used to ensure the complete eradication of bacteria. In addition to killing bacteria, these agents, primarily phenolic compounds, were also highly irritating to the periradicular tissues.^1^,^2^ Overzealous use of these medicaments led to postoperative complications that were erroneously identified as persistent periradicular infections. Hence, this led to the inappropriate and excessive use of antibiotics to control infections. Ultimately the deleterious effects of these medicaments were identified^1^ and their routine clinical use was discontinued. This led to one of the two course of treatment either treat the root canal in one visit or seek an intracanal medicament that does not injure the periradicular tissues.

Those who believe that successful root canal treatment can be completed in one visit have rationale in literature. Studies concerning postoperative pain^3^-^6^ as well as healing rates^7^-^9^ shows the treatment outcome to be similar whether completed in one or multiple visits. In addition to this, treatment in one visit offers many advantages. This decreases the number of operative procedure including additional anesthesia, gingival trauma from rubber dam application as well as eliminating the risk of inter appointment leakage through temporary restoration. It is less time consuming resulting in less cost to the patients.

Proponents of multiple visit procedures contend that antimicrobial property of inter appointment calcium hydroxide placement is required to ensure successful perradicular healing,^10^-^12^ although predictable levels of bacterial reduction via refined cleaning and shaping techniques is one appointment may negate this need.^13^

Furthermore, when flare-ups occur during multiple-visit procedures, they can be addressed prior to obturation.^7^ This is not an option in a single-visit treatment regimen. When flare-ups occur, non-surgical re-treatment or surgical intervention is usually necessary.

The purpose of the study was to determine clinical success rate of single visit verses multiple visit root canal treatment in cariously exposed vital primary molars.

## INDICATIONS FOR SINGLE-VISIT TREATMENT

### Isolation and Sealing Problems

One of the main objectives when endodontics is performed in multiple visits is the difficulty of effectively sealing off the root canal system from the oral cavity between visits. Although this aim may be easily obtained in most cases, there are certain situations in which a one-visit procedure can be used to eliminate the potential problem of interappointment contamination and/or flare-up.

Teeth with subgingival breakdown; coronal walls missing; and with full coverage that have decay below the margins of their finished restorations would all fall into this category.^14^

### Anterior Esthetic Problems

Cases falling into this category would be maxillary anterior teeth involved in trauma that has resulted in a horizontal fracture of the crown at the gum line. These cases are probably the most frequently treated teeth in one-visit. Therefore, isolation and sealing problems are solved and an esthetic temporary crown can be placed rapidly and retained by securing the crown to a temporary post placed into the space left in the root canal of the treated tooth.^14^

### Restorative Considerations

Cases that fall into this category require endodontic treatment for restorative reasons and not because they have pathologic pulp tissue that must be removed or because of pulp exposures. Examples would include: teeth to be used as overdenture abutments; mandibular anterior teeth to be cut down for full jacket crowns; teeth with severe coronal breakdown that cannot possibly retain a restoration because of the loss of tooth structure; and teeth that require preparation that would result in pulp exposure in order to get them into a certain desired alignment for the construction of a specifically designed restoration.^14^

### Vital Pulp Exposure and Symptomatic Pulpitis

Teeth containing vital pulps that fit into this category are those with pulp exposures caused by trauma, caries, or mechanical reasons and teeth that exhibit clinical symptoms to heat or cold stimuli but not percussion.^14^

## MATERIAL AND METHODS

A sample of 40 children in age group of 4 to 7 years visiting to Department of Pedodontic for dental treatment at Govt. Dental College, Rohtak, were included in this study after receiving permission from their parents. 40 teeth which were cariously exposed showing no sign of abnormal mobility, swelling or sinus tract formation and requiring pulpectomy were selected for study. 

These were randomly into two equal groups.

*Group I:* Single visit treatment group

*Group II:* Multiple visit treatment group

Endodontic therapy in each case was carried out under local anesthesia and rubber dam isolation. The pulp was extirpated and diagnostic radiographs were made to determine working length. Biomechanical preparations were done using 2.5% sodium hypochlorite as root canal irrigant.

In single visit group, after biomechanical preparation, root canals were dried using absorbent paper points and root canals were filled with thick mix of zinc oxide eugenol using engine driven lentulo spirals. Access cavities were sealed with silver amalgam/glass ionomer cement after obturation.

In multiple visit group, access was gained and after biomechanical preparation, root canals were dried and filled with calcium hydroxide powder mixed with normal saline and access cavities were sealed with zinc oxide eugenol cement. After 7 days, calcium hydroxides dressing were removed with reamers and normal saline as irrigant (calcium hydroxide dissolved in this solution). The root canals were dried using absorbent paper points and obturated with zinc oxide eugenol cement using engine driven lentulo-spirals. Access cavities were sealed with silver amalgam/glass ionomer cement.

Recall visits were carried out after one week, one month and three months and six months. Success and failure of treatment was evaluated according to criteria laid down by Gutmann (1992)^15^ (Table 1).

**Table 1:** TABLE 1: Guidelines for clinical and radiographic success (adapted from Gutmann 1992)

		*Success*		*Questionable*		*Failure*
Clinical		No tenderness topercussion or palpation		Sporadic vague symptomology often not reproducible		Persistent subjective symptoms
		Normal mobility		Pressure sensation or feeling of fullness		Recurrent sinus tract or swelling
		No sinus tract formation		Low grade discomfort following percussion, palpation or chewing		Predictable discomfort to percussion or palpation
		Tooth function		Discomfort when pressure is applied by the tongue		Evidence of irreparable tooth fracture
		No sign of infection or swelling		Superimposed sinusitis with a focus on the treated tooth		Excessive mobility or progressive periodontal breakdown
		No evidence of subjective discomfort		Occasional need for analgesics to relieve minimal discomfort		Inability to function on the tooth
Radiographic		Normal to slightly thickened periodontal ligament space (< 1 mm)		Increased periodontal ligament space (> 1 mm and < 2 mm)		Increased width of periodontal ligament space (> 2 mm)
		Elimination of previous rarefaction		Stationary rarefaction or slight repair evident		Lack of osseous repair within rarefaction or increased rarefaction
		Normal lamina dura in relation to adjacent teeth		Increased lamina dura in relation to adjacent teeth		Lack of new lamina dura
		No evidence of resorption		Evidence of resorption		Presence of osseous rarefactions in periradicular areas where previously none existed
		Dense, three dimensional obturation of canal space extending to cementum dentin junction (1 mm from apex)		Voids in obturation density		Visible patent canal space &ndash; unfilled or significant voids in obturation
				Extension of filling material beyond anatomic apex		Excessive overextension with voids in apical third active resorption coupled with other radiographic signs of failure		

## RESULTS (TABLE 2, FIG. 1)

### In Single Visit Group

One Week

Two patients came with swelling and pain after two days of obturation. Antibiotics and analgesics were prescribed to him. Symptoms disappeared after seven days.

One Month

One of the two patient reported with postoperative complication , reported with intraoral sinus. Filling material was removed from primary molar and patient was treated according to multiple visit group regimen.

Three Month

Remaining all patients were asymptomatic.

Six Months

Remaining all patients were asymptomatic.

### In Multiple Visit Group

One Week

All patients were asymptomatic.

One Month

All patients were asymptomatic.

Three Months

All patients were asymptomatic.

Six Months

Remaining all patients were asymptomatic.

**Table 2:** TABLE 2: Results

*Number of sample*				*Group I*		*Group II*
				20		20
Success percentage		1 week		90		100
		1 month		95		100
		3 months		95		100
		6 months		95		100
Failure percentage		1 week		10		0
		1 month		5		0
		3 months		5		0
		6 months		5		0

To compare the number of success with failure in two groups we apply Fisher Exact test. In the first week 18 out of 20 patients gives successful result in group I where in group II all 20 patients gives successful result. This gives the non significant difference in the results of two groups with test value 0.53 (p-value = 0.48) (if this p-value is <0.05 then we can say that there is significant difference in the outcomes of the groups).

After one month group I, 18 out of 20 patients show successful result, whereas in group II all 20 patients give successful result. This failure of two patients in group I is not significantly different from group II, having test value 0.53 (p-value = 0.48)

Since there is single patient which report negative result in group I against all 20 successful patients in group II after three month. This also shows nonsignificant difference in both the treatments having test value 0.001 and p-value 0.99.

After six months one patient from each group was not reported. So excluded from the analysis and from remaining 19 patients only single gives negative response in group I against all 19 positive responses in group II. This also shows nonsignificant differences with t-value 0.001 and p-value 0.99.

 In all we can say that there is no significant difference in treatments results. 

**Fig. 1:**
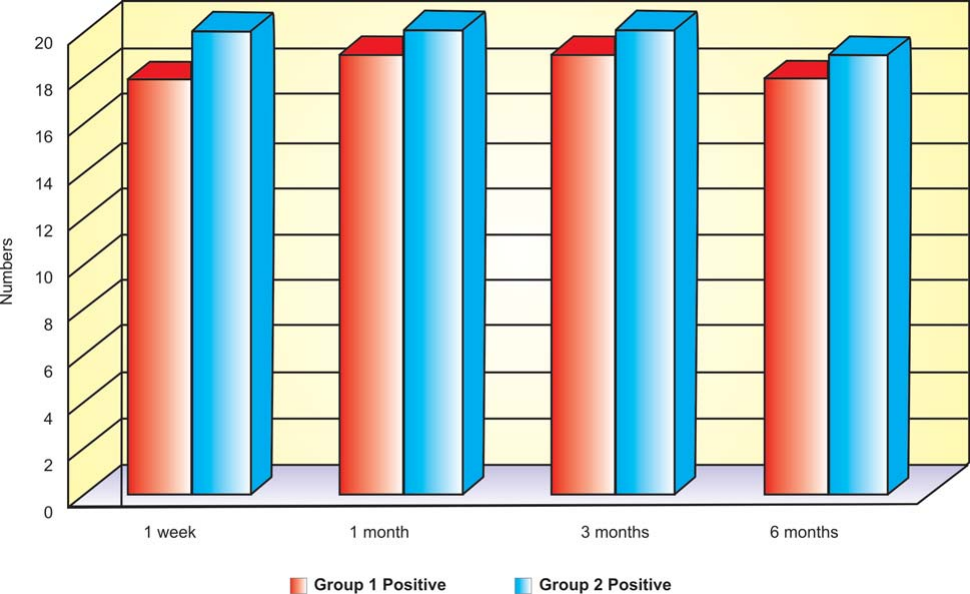
Positive results cases in two groups

## DISCUSSION

In present study, patients in the age group of 4-7 years were selected because root formation of primary molars has been completed up to 4 years of age and root resorption of primary molars has not been started up to 7 years of age. Further patients were followed-up for six months so that any postoperative complications like pain, swelling, sinus formation can be evaluated. Primary molars which were cariously exposed, were selected for study.

Because in an infected vital pulp due to carious exposure, the infection is normally found only at the wound surface, where it has resulted in a localized inflammatory response. This means that in most apical portion of pulp tissue, micro organisms are usually not present. Root canal treatment in such cases is carried out on presumption that the pulpal condition is irreversible and may lead to tissue break down and subsequent root canal infection.^16^ Root canal treatment in such cases can be carried out by single visit or multi visit therapy. So in present study equal criteria of case selection had been used for both groups.

Most of the studies comparing the success rate of endodontic therapy performed in one or more sessions have been based on poorly defined criteria of evaluation. Pekruhn^8^ in a very large study, but without defined criteria, concluded that there were significantly fewer failures in the two-visit treatment group than in the one-visit treatment group. On the other hand, in a very controlled clinical study, Sjogren et al^17^ investigated the role of infection on the outcome of one-visit treatment after a follow-up period of five years. Success was reported for 94% of the infected root canals associated with periradicular lesions that yielded negative culture, whereas in the samples which yielded positive culture prior to root filling, the success rate of treatment was 68%; thus, stressing the use of intracanal medication in infected cases (Table 3).

In another well-controlled study, Trope et al^18^ investigated radiographic healing of teeth with periradicular lesions treated in one or two visits. In the two-visit group, root canals were medicated with calcium hydroxide for at least one week. After a one-year follow-up evaluation, the additional disinfecting action of calcium hydroxide resulted in a 10% increase in healing rates. This difference should be considered clinically important.

Katebzadeh and associates^19^,^20^histologically and radiographically compared periradicular repair after treatment of infected root canals of dogs performed in one or two sessions. They reported better results for the twovisit treatment in which calcium hydroxide was used as an intra canal disinfectant for one week.

In our study, the success rate in both techniques was almost equal with one patient reported with postoperative complications in single visit group. The results obtained are similar to that obtained by Rudner and Oliet (1981), 21Trope et al (1999), 18 Katebzadeh et al (2000).^20^ They also reported that success rate of multiple visit technique is slightly more than single visit technique.

The probable reason for this difference may be the fact that bacterial load can be decreased by additional dressing of calcium hydroxide as intra canal medicament between the appointments. Calcium hydroxide has been widely used in endodontics. Currently, this chemical substance is acknowledged as one of the most important antimicrobial dressing during endodontic therapy. 22 Most endodontic pathogens are unable to survive in a highly alkaline environment such as that of calcium hydroxide. Therefore, several bacterial species commonly found in infected root canals are eliminated after a short period when in direct contact with this substance.^23^ The anti-microbial activity of calcium hydroxide is related to the release of hydroxyl ions in an aqueous environment. Direct contact experiments in vitro show that a 24-hour contact period is required for complete killing of enterococci.^24^ In clinical experimentation, one week of intracanal dressing has been shown to safely disinfect a root canal system. 25 A study of 42 patients found that sodium hypochlorite irrigation reduced the bacteria level by only 61.9%, but use of calcium hydroxide in canals for one week resulted in 92.5% reduction.^26^

In addition to killing bacteria, calcium hydroxide has ability to hydrolyse the lipid moiety of bacterial lipopolysaccharide (LPS), thereby inactivating the biologic activity of lipopolysaccharide (LPS) and reducing its effect.^27^,^28^ This is very desirable effect because dead cell wall material remain after the bacteria have been killed and can continue to stimulate inflammatory response in the periradicular tissue.

**Table 3:** TABLE 3: Studies evaluating healing of single visit and multiple visit root canal treatment

*Study*		*Model*		*Cleaning*						*%*				
				*and shaping*		*% age*		*Healing*		*success*		*Intracanal*		*%*
				*technique*		*NaOCl*		*termination*		*1V*		*medicament*		*success MV*
Katebzadeh		Dog		To ISO 45		-		Radiographic		35.3		CaOH_2_		36.8
et al 2000^20^														
Trope et al		Human		Not indicated		2.5		Radiographic		80.0		CaOH_2_		81.0
(1999)^18^														
Rudner and		Human		Hand		2.3 w/3%		Clinical and		89.7		Not		91.1
Oliet (1981)^21^				instrumentation		H_2_O_2_		radiographic				specified		
Soltanoff		Human		Hand										
(1978)^7^				instrumentation		—		Radiographic		85.0		Not specified		88.0
Ashkenaz		Human		Hand instru-		5.0		Clinical and		97.0		—		—
(1979)^29^				mentation				radiographic						
				step back										
Oliet (1983)^5^		Human		Not indicated		5.0		Clinical and radiographic		89.0		Not specified		89.0

**Table 4:** TABLE 4: Comparative studies on the incidence of postoperative pain after one-visit endodontics

*Investigator*		*Tooth*		*Pulpal*		*Total*		*Severity (one-visit)*				*Total*		*Severity (one-visit)*		
		*group*		*status*		*cases*						*cases*				
								*None-slight*		*Mod-severe*				*None-slight*		*Mod-severe*
Ferranti P^30^		AP		N-V		178		162 (91%)		9 (9%)		162		156 (96.2%)		6 (3.8%)
Fox J,		AP		V N-V		247		222 (90%)		25 (10%)				Not studied		
Atkinson JS,																
Dinin PA,																
et al^3^																
O’Keefe EM^31^		AP		V N-V		55		54 (98%)		1 (2%)		77		70 (91%)		7 (9%)
Soltanoff W^7^		AP		V N-V		89		71 (81%)		17 (19%)		193		166 86(%)		27 (14%)
Ashkenaz PJ^29^		Single		V		195		187 (96%)		8 (4%)				Not studied		
		rooted AP															
Rudner WL,		AP		V N-V		98		87 (88.5%)		11 (11.5%)		185		164 (88.5%)		21 (11.5%)
and Oliet, S.^21^																
Mulhern JM,		Single		N-V		30		23 (76.7%)		7 (23.3%)		30		22 (73.3%)		8 (26.7%)
Patterson SS,		rooted AP														
Newton CW,																
et al^4^																
Oliet S^31^		AP		V N-V		264		236 (89%)		28 (11%)		123		115 (93.4%)		8 (16.6%)
Roane JB,		AP		V N-V		250		212 (84.8%)		38 (15%)		109		75 (68.8%)		34 (31.2%)
Grimes EW^32^																

Abbreviations: A = Anteriors; P = Posteriors; V = Vital; N-V = Non-vital.

Note: Severity: None to slight = patient took no analgesic, or a non-narcotic analgesic to relieve pain.

Moderate to severe = patient took narcotic analgesic for relief of pain.

Postoperative pain after non-surgical root canal treatment has been reported to range from app. 3 to 50% 4,33,34 (Table 4). In our study two patients reported with post operative pain in single visit group and no patient reported with pain in multiple visit group.

Ferranti^30^ reported relatively low incidence of server pain following single visit procedure. O’Keefe^31^ found no significant difference in postoperative pain experience by his patient following single visit or multiple visit root canal treatment. Soltanoff 7 used a random selection of cases treated during 20 years period to compare single and multiple visit treatment by degree of postoperative pain experienced found following single visit treatment more than 50% of his patient experienced pain. Roane et al^32^ reported 2:1 higher frequency of pain following treatment completed in multiple visit as compared to that reported for those treated with single visit. Mulhern et al.^4^ concluded that there was no significant difference in the incidence of postoperative pain between one-visit and multiple-visit endodontic treatment of asymptomatic pulpal necrosis. Moreover, pain associated with root canal therapy is poor indicator of pathosis and even more unreliable predictor of long term success.^35^

## CONCLUSION

We conclude that multiple visit and single visit root canal treatment demonstrated almost equal success. However, long term follow-up and big sample size are required to further corroborate the findings of this study. Most important aspect for success in pulpectomy cases is the indication of each case and then its subsequent treatment, be it multiple or single visit root canal treatment.

Briefly, in cases of vital pulp, a single-visit treatment should be used whenever possible. This is based on the fact that the pulp is only superficially infected and the root canal is free of bacteria, provided the aseptic chain is maintained during the intra canal procedures. Therefore, there is no apparent reason not to treat vital pulps in a single visit. Conversely, if the pulp is necrotic and/or associated with a periradicular disease, there is ample evidence that the root canal system is infected.^36^ In these cases, the root canal system should ideally be cleaned and shaped, an intracanal medication placed, and the canal filled at a second appointment.

One visit endodontic should be viewed as a procedure that supplements and complements total patient care as it relates to endodontics and not as a technique that is going to totally replace multivisit procedures. Both single and multivisit treatments should be viewed as part of a total endodontic treatment spectrum, with the choice of one over the other being determined by the circumstances surrounding each individual case. The practitioner should not routinely apply one technique to all situations, but rather evaluate the circumstances peculiar to each particular case and then choose the technique tat best fits those circumstances. However, when doubt exists, the multiple visit procedure should be performed. Thus, the clinician will be most effectively utilizing his time in delivering the best possible endodontic service available to the patient.^14^

## References

[1] Messer HH, Feigal RJ (1985). A comparison of the antibacterial and cytotoxic effects of parachlorophenol.. J Dent Res.

[2] Koontongkaew S, Silapichit R, Thaweboon B (1988). Clinical and
laboratory assessments of camphorated monochlorophenol in
endodontic therapy.. Oral Surg Oral Med Oral Pathol.

[3] Fox J, Atkinson JS, Dinin AP, Greenfield E, Hechtman E, Reeman CA, Salkind M, Todaro CJ (1970). Incidence of pain following
one-visit endodontic treatment.. Oral Surg Oral Med Oral Pathol.

[4] Mulhern JM, Patterson SS, Newton CW, Ringel AM (1982). Incidence
of postoperative pain after one appointment endodontic
treatment of asymptomatic pulpal necrosis in single-rooted teeth.. J Endod.

[5] Oliet S (1983). Single-visit endodontics: a clinical study.. J Endod.

[6] Eleazer PD, Eleazer KR (1998). Flare-up rate in pulpally necrotic molars
in one-visit versus two-visit endodontic treatment.. J Endod.

[7] Soltanoff W (1978). A comparative study of the single-visit and the
multiple-visit endodontic procedure.. J Endod.

[8] Pekruhn RB (1986). The incidence of failure following single-visit endodontic therapy. J Endod.

[9] Weiger R, Rosendahl R, Lost C (2000). Influence of calcium hydroxide
intracanal dressings on the prognosis of teeth with endodontically
induced periapical lesions.. Int Endod J.

[10] Bystrom A, Sundqvist G (1985). The antibacterial action of sodium hypochlorite and EDTA in 60 cases of endodontic therapy.. Int Endod J.

[11] Sjogren U, Figdor D, Persson S, Sundqvist G (1997). Influence of infection
at the time of root filling on the outcome of endodontic treatment
of teeth with apical periodontitis.. Int Endod J.

[12] Trope M, Delano EO, Orstavik D (1999). Endodontic treatment of
teeth with apical periodontitis: single vs. multivisit treatment.. J Endod.

[13] Card SJ, Sigurdsson A, Orstavik D, Trope M (2002). The effectiveness
of increased apical enlargement in reducing intracanal bacteria.. J Endod.

[14] Ashkenaz PJ (1984). One-visit endodontics.. Dent Clin North Am.

[15] Gutmann JL (1992). Clinical, radiographic, and histologic perspectives
on success and failure in endodontics.. Dent Clin North Am.

[16] Trope M, Bergenholtz G (2002). Microbiological basis for endodontic
treatment: can a maximal outcome be achieved in one visit?. Endod
Topics.

[17] Sjögren U, Figdor D, Persson S, Sundqvist G (1997). Influence of infection
at the time of root filling on the outcome of endodontic treatment
of teeth with apical periodontitis.. Int Endod J.

[18] Trope M, Delano EO, Orstavik D (1999). Endodontic treatment of
teeth with apical periodontitis: single vs. multivisit treatment.. J Endod.

[19] Katebzadeh N, Hupp J, Trope M (1999). Histological periapical repair
after obturation of infected root canals in dogs.. J Endod.

[20] Katebzadeh  N, Sigurdsson A, Trope M (2000). Radiographic evaluation
of periapical healing after obturation of infected root canals: an
in vivo study.. Int Endod J.

[21] Rudner WL, Oliet S (1981). Single-visit endodontics: a concept and a
clinical study.. Compend Contin Educ Dent.

[22] Farhad A, Mohammadi Z (2005). Calcium hydroxide: a review.. Int
Dent J.

[23] Bystrom A, Claesson R, Sundqvist G (1985). The antibacterial effect of camphorated paramonochlorophenol, camphorated phenol and calcium hydroxide in the treatment of infected root canals.. Endod Dent Traumatol.

[24] Safavi KE, Spangberg LS, Langeland K (1990). Root canal dentinal tubule
disinfection.. J Endod.

[25] Sjogren U, Figdor D, Spangberg L, Sundqvist G (1991). The antimicrobial
effect of calcium hydroxide as a short-term intracanal dressing.. Int Endod J.

[26] Shuping GB, Orstavik D, Sigurdsson A, Trope M (2000). Reduction of
intracanal bacteria using nickel-titanium rotary instrumentation
and various medications.. J Endod.

[27] Safavi KE, Nichols FC (1994). Alteratoin of biological properties of
bacterial lipopolysaccharide by calcium hydroxide treatment.. J Endod.

[28] Safavi KE, Nichols FC (1993). Effect of calcium hydroxide on bacterial
lipopolysaccharide.. J Endod.

[29] Ashkenaz PJ (1979). One-visit endodontics--a preliminary report.. Dent
Surv.

[30] Ferrangi P (1959). Treatment of root canals of infected teeth in one
appointment: a report of 340 cases.. Dent Dig.

[31] O'Keefe EM (1976). Pain in endodontic therapy: preliminary study.. J Endod.

[32] Roane JB, Dryden JA, Grimes EW (1983). Incidence of postoperative
pain after single and multiple-visit endodontic procedures.. Oral
Surg Oral Med Oral Pathol.

[33] Walton R, Fouad A (1992). Endodontic interappointment flare-ups: a
prospective study of incidence and related factors.. J Endod.

[34] Torabinejad M, Cymerman JJ, Frankson M, Lemon RR, Maggio JD, Schilder H (1994). Effectiveness of various medications on
postoperative pain following complete instrumentation.. J Endod.

[35] Taintor JF, Langeland K, Valle GF, Krasny RM (1981). Pain: a poor
parameter of evaluation in dentistry.. Oral Surg Oral Med Oral
Pathol.

[36] Siqueira JF Jr. (2001). Strategies to treat infected root canals.. J Calif
Dent Assoc.

